# Responsive 3D Printed Microstructures Based on Collagen Folding and Unfolding

**DOI:** 10.1002/smll.202408597

**Published:** 2024-11-27

**Authors:** Philipp Mainik, Camilo Aponte‐Santamaría, Magdalena Fladung, Ronald Ernest Curticean, Irene Wacker, Götz Hofhaus, Martin Bastmeyer, Rasmus R. Schröder, Frauke Gräter, Eva Blasco

**Affiliations:** ^1^ Institute for Molecular Systems Engineering and Advanced Materials (IMSEAM) Heidelberg University 69120 Heidelberg Germany; ^2^ Organic Chemistry Institute (OCI) Heidelberg University 69120 Heidelberg Germany; ^3^ Heidelberg Institute for Theoretical Studies (HITS) 69118 Heidelberg Germany; ^4^ Cell and Neurobiology Zoological Institute Karlsruhe Institute of Technology (KIT) 76131 Karlsruhe Germany; ^5^ BioQuant Heidelberg University 69120 Heidelberg Germany; ^6^ Institute for Biological and Chemical Systems – Biological Information Processing (IBCS‐BIP) Karlsruhe Institute of Technology (KIT) 76344 Karlsruhe Germany; ^7^ Interdisciplinary Center for Scientific Computing (IWR) Heidelberg University 69120 Heidelberg Germany

**Keywords:** 4D printing, extracellular matrix, self‐assembly, stimuli‐responsive materials, two‐photon laser printing

## Abstract

Mimicking extracellular matrices holds great potential for tissue engineering in biological and biomedical applications. A key compound for the mechanical stability of these matrices is collagen, which also plays an important role in many intra‐ and intercellular processes. Two‐photon 3D laser printing offers structuring of these matrices with subcellular resolution. So far, efforts on 3D microprinting of collagen have been limited to simple geometries and customized set‐ups. Herein, an easily accessible approach is presented using a collagen type I methacrylamide (ColMA) ink system which can be stored at room temperature and be precisely printed using a commercial two‐photon 3D laser printer. The formulation and printing parameters are carefully optimized enabling the manufacturing of defined 3D microstructures. Furthermore, these printed microstructures show a fully reversible response upon heating and cooling in multiple cycles, indicating successful collagen folding and unfolding. This experimental observation has been supported by molecular dynamics simulations. Thus, the study opens new perspectives for designing new responsive biomaterials for 4D (micro)printing.

## Graphical abstract

Two‐photon 3D laser printing of free‐standing, fibrillar, and biocompatible 3D collagen microstructures is presented using a room temperature stable, easily accessible ink and a commercial setup. In addition, it is shown for the first time that 3D‐printed collagen microstructures exhibit reversible response behavior, resulting in large volume shrinkage upon heating and cooling in multiple cycles.

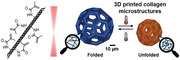

## 1 Introduction

Natural extracellular matrices (ECMs) have served as an excellent source of inspiration for the design of cell scaffolds for biological and medical applications.^[^ ^1^, ^2^ ^]^ ECM‐mimicking biomaterials and their processing in defined 3D structures have been gaining more and more interest in recent years.^[^ ^3^ ^]^ Various advanced manufacturing techniques such as direct ink writing and light‐based techniques relying on photopolymerization processes have been employed for these purposes.^[^ ^4^, ^5^, ^6^, ^7^, ^8^ ^]^ Recently, two‐photon 3D laser printing, also known as multi‐photon lithography or direct laser writing, has attracted much attention. This 3D printing technology offers precise control in manufacturing on the micro‐ to nanometer scale, being highly suitable for the preparation of (single) cell 3D scaffolds with sub‐cellular resolution.^[^ ^9^, ^10^ ^]^ In particular, polysaccharides (e.g., methacrylate chitosan or hyaluronic acid), as well as proteins (e.g., bovine serum albumin), cover a large range of biocompatible and easily accessible biomaterials exploited in the field.^[^ ^11^, ^12^, ^13^, ^14^, ^15^, ^16^, ^17^, ^18^, ^19^, ^20^, ^21^, ^22^, ^23^, ^24^ ^]^ These previously described bio‐derived materials for two‐photon 3D laser printing were mainly employed as non‐responsive, structural hydrogels for the preparation of biocompatible cell scaffolds or microswimmers. Printability of these biomaterials is usually achieved either by functionalizing hydroxy or amine side groups of the biopolymers with photocrosslinkable thiol‐ene or (meth)acrylate groups or by using photosensitizers which often imply the use of high doses.^[^ ^25^ ^]^

High‐resolution 3D printing of collagen is of great interest since it is the major structural component in the ECM and hence, collagen microstructures are close to mimicking the ECM.^[^ ^26^ ^]^ In ECMs, collagen provides exceptional mechanical stability while maintaining perfusive properties.^[^ ^27^, ^28^ ^]^ The mechanical stability originates from the assembly of collagen into triple helices which crosslink and form higher‐order assemblies to fibrils and fibers. First studies in the direction of microprinting of ECMs employed native collagen type I in the presence of photosensitizers such as riboflavin or rose bengal to create 2D patterns by crosslinking the collagen.^[^ ^29^, ^30^, ^31^, ^32^, ^33^ ^]^ Custom‐made set‐ups were necessary due to the high laser doses required, limiting its application in fabricating 3D scaffolds. One way to overcome these drawbacks is a chemical modification of collagen. This approach enabled efficient photo‐crosslinking of the collagen and therefore significantly lower energy doses are required for printing, making it more attractive for biomedical applications. For example, Tytgat et al. 3D printed isobornene‐ and thiol‐functionalized recombinant collagen to encapsulate cells.^[^ ^34^ ^]^ In another work, Shrestha et al. microprinted commercially accessible collagen type I methacrylamide (ColMA) for culturing retinal cells.^[^ ^35^ ^]^ In the latter, the authors achieved improved printability compared to native collagen, but cooling the ink during printing was necessary to avoid its precipitation. In addition to these technical limitations, it remains unclear if the printing process allows collagen self‐assembly as known in ECMs, as the retention of the secondary structure of collagen after 3D printing has not been demonstrated yet.

**Figure 1:**
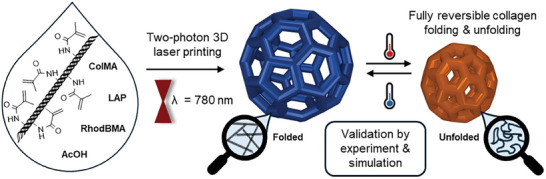
Two‐photon 3D laser printing of collagen methacrylamide (ColMA) in the presence of a water‐soluble photoinitiator (LAP), fluorescent dye (RhodBMA), and acetic acid (AcOH) at room temperature. The microprinted structures showed a fully reversible temperature‐responsive behavior based on collagen folding and unfolding. The experimental observation was supported by molecular dynamics simulations.

In this work, we present a robust and facile methacrylamide‐functionalized collagen‐based printable system that is stable at ambient conditions and can be printed with a commercially available setup. This approach offers fast microfabrication of biocompatible arbitrary 3D collagen structures increasing the versatility and applicability of current efforts. Moreover, we show temperature‐responsive reversible collagen folding and unfolding not only in the ink but also 3D printed microstructures for the first time, leading to large and reproducible volume changes (**Figure** **1**). The fully reversible temperature‐dependent response is to the best of our knowledge the first demonstration of responsive self‐assembly of collagen in (3D printed) materials.

## 2 Results & Discussion

### 2.1 3D Microprinting of Collagen

The first step toward achieving an easily accessible approach for high‐resolution 3D printing of collagen was the design of a printable formulation (ink) that is stable and compatible with two‐photon 3D laser printing (Figure 1). We selected ColMA as a suitable starting material since it is easily accessible either commercially or by methacrylation of native collagen and exhibits increased thermal stability by reversibly forming physical gels between 4 and 34 °C.^[^ ^36^, ^37^ ^]^ In order to overcome the room temperature instability of previously reported collagen inks, the ColMA was dissolved in an acetic acid medium (0.02 M) in which native collagen exhibits increased thermal stability.^[^ ^38^, ^39^, ^40^ ^]^ Before printing, the preservation of the collagen triple‐helix structure, i.e., folded collagen, was verified by UV circular dichroism (UV‐CD) spectroscopy (Figure S1, Supporting Information). The ColMA solution showed two characteristic bands at 200 and 220 nm resembling reported characteristic native collagen folding bands.^[^ ^26^, ^41^, ^42^ ^]^ Thus, the collagen methacrylamide type I ink (collagen ink) was prepared by dissolving ColMA in a concentration of 8 mg mL^−1^ in 0.02 m acetic acid and adding lithium phenyl‐2,4,6‐trimethyl‐benzoylphosphinate (LAP) (35 mg mL^−1^), a water‐soluble and biocompatible photoinitiator. Additionally, a fluorescence dye, methacryloxyethyl thiocarbamoyl rhodamine B (RhodBMA), was added to facilitate subsequent imaging of the 3D printed microstructures using confocal fluorescence microscopy. The methacrylate group enabled chemically covalent incorporation of the fluorescent dye in the printed structure. The collagen ink (pH 3.8) was characterized for its viscosity by rotational rheology and optical properties by refractometry as well as UV and infrared spectroscopy (Figures S3–S6, Supporting Information). Furthermore, we investigated the morphology of our collagen ink by cryo‐transmission electron microscopy and found fibrils exhibiting thicknesses of 12.3 nm ± 2.5 nm (Figures S7,S8, Supporting Information). Importantly, the collagen ink was stable for several weeks at room temperature without showing precipitation or any significant change in printability.

**Figure 2:**
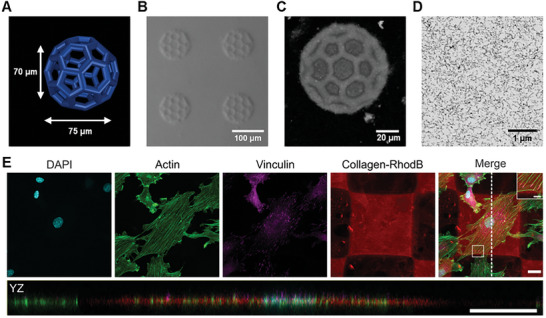
Two‐photon 3D laser printing of collagen ink at room temperature. A) Model of empty buckyball as complex 3D microstructure with overhanging features in dimensions of 75 µm × 75 µm × 70 µm. B) Optical microscopy image acquired with CDIC contrast of micrometric buckyballs which were successfully printed with a laser power of 42 mW and scanning speed of 20 mm^−1^s^−1^. C) Top view (XY) on volumetric 3D reconstruction showing the porous free‐standing features of microprinted collagen buckyballs acquired with confocal fluorescence microscopy. D) Large field of view scanning electron microscopy image of printed collagen nanostructure after embedding and sectioning showing the distribution of fibrils in the ultrathin section. The printed structure is composed of 13.2 nm ± 2.4 nm thick fibrils. E) Confocal images of immunocytochemical staining of rat embryonic fibroblast (REF) cells cultured on 3D laser‐printed collagen blocks. Cells adhered to the printed scaffold (red) and exhibited an undisturbed actin organization (green). The magnification of the indicated area shows that focal adhesions, as visualized by Vinculin‐staining (magenta), formed on the RhodBMA‐labelled collagen blocks (red). Cells adhered to the top of the scaffolds as shown by the YZ‐projection. The projection plane is indicated by a white dotted line in the merged image. Scale bars: 20 µm, magnification: 5 µm.

The printability of the collagen formulation was tested using a commercial two‐photon 3D laser printing set‐up (Photonic GT2, Nanoscribe GmbH) with a 25× objective. As a first step, 3D cubic lattice microstructures with dimensions of 100 µm × 100 µm × 70 µm were printed with varying printing parameters such as laser powers ranging from 21 to 42 mW and scanning speeds from 20 to 60 mm^−1^s^−1^. The broad printability window for printing these microstructures is depicted in Figure S9 (Supporting Information). After removal of unpolymerized ink washing first with a 0.02 m acetic acid solution and subsequently with water (HPLC grade), the structures retained their printed dimensions. The 3D‐printed collagen microstructures were stable at pH conditions between 2.6 and 10.8 (see Figure S10, Supporting Information). Furthermore, the fabrication of complex hollow 3D geometries, such as micrometric buckyballs (**Figure** **2**A) with a diameter of ≈75 µm was successfully achieved, proving the good printability and versatility of the new collagen ink. First, the printed collagen was imaged by optical microscopy using contrast methods such as phase or circular differential interference contrast due to the high content of water in the 3D microstructures (Figure 2B). Additionally, confocal microscopy was employed to analyze the printed buckyballs in 3D (Figure 2C). A 3D reconstruction using experimental data proved the hollow features as expected from the model with good accuracy (see Figure S11, Supporting Information).

The precision of the printing process was further experimentally quantified by printing single parallel lines and analyzed using confocal microscopy. Resolved lines have been observed when printing with a distance of 1.5 µm between them (see Figure S12, Supporting Information). The minimum feature size was determined as 460 nm ± 70 nm by measuring the thickness of five separate lines.

As a next step, we aimed to analyze the internal nanostructure of the microprinted collagen by using electron microscopy to see how the fibrils in the ink translate into the printed collagen. The high‐water content of the collagen microstructures (>99% in the collagen ink) led to an irreversible collapse upon simple drying. Therefore, we have first replaced the water stepwise with ethanol and subsequently, acetone to perform either critical point drying or embedding the printed microstructures in epoxide resin. Critical point drying led to a partial collapse of the microstructures (see Figure S13, Supporting Information), so we focused on employing an embedding protocol. For this purpose, we prepared 3D blocks of 50 µm × 50 µm × 50 µm (laser power 42 mW and scanning speed of 25 mm^−1^s^−1^). The 3D‐printed collagen micrometric blocks were embedded and sectioned, and the cross‐sections were imaged by scanning electron microscopy (SEM). SEM images indicated a fibrillar network (see Figure 2D for a large field of view and Figures S14,S15, Supporting Information for the zoom‐in). The average diameter of the fibrils in the printed collagen was found to be 13.2 nm ± 2.4 nm (Figure S16, Supporting Information). In addition to SEM, we performed TEM of 70 nm thick sections. The embedded printed collagen exhibited an internal fibrillar nanostructure (Figure S17, Supporting Information) as already suggested by SEM. The diameter obtained by TEM was 12.6 nm ± 2.3 nm (Figure S18, Supporting Information) and was found to be similar to the determined average from SEM. Comparing the fibrils in the ink with the fibrils observed in the microprinted collagen suggested that there is no apparent influence on the fibrils during the printing process (see Table S1, Supporting Information).

Next, biocompatibility, i.e., cell viability and immunocytochemical analysis, was addressed by culturing rat embryonic fibroblast cells on the 3D‐printed collagen scaffolds. In detail, cells were seeded on arrays of 10 × 10 printed squares (100 µm × 100 µm, height 5 µm) as shown in Figure S19 (Supporting Information). For cell viability, live and dead cells were labeled with Calcein‐AM and ethidium homodimer (EthD1) after 24 h, respectively. Additionally, cell nuclei were visualized with Hoechst. The obtained fluorescence images are depicted in Figure S20 (Supporting Information). Throughout three independent experiments, the majority of cells appeared positive for the live stain and negative for the dead stain. Statistical analysis with a total number of N = 473 cells verified > 99% of cells to be alive (Figure S21, Supporting Information). Immunocytochemical staining was performed to verify the adhesion and unaltered cytoskeletal organization of the cells. As depicted in Figure 2E and Figure S22 (Supporting Information), focal adhesions formed normally as shown by the vinculin staining and the actin skeleton appears to be undisturbed. The orthogonal projection in YZ shows that the cells are well spread and adhered to the top of the scaffold. These results indicate that the collagen ink did not significantly impact cell viability and did not interfere with the organization of the actin cytoskeleton or the formation of focal adhesions. Hence, the herein‐used collagen ink exhibits good biocompatibility.

### 2.2 Response Study of the Collagen 3D Microstructures

Once we had characterized the internal fibrillar nanostructure of the 3D‐printed collagen, we used temperature as a stimulus to study the response. It is well‐known that heating of collagen leads to uncoupling of the triple helices.^[^ ^26^, ^37^ ^]^ Therefore, we were interested in exploiting this effect in 3D‐printed collagen. First, we verified the temperature‐induced unfolding of the collagen ink by UV‐CD spectroscopy (see Figures S23–S25, Supporting Information). Upon heating above 37 °C, which is known to be the temperature range for unfolding or denaturation of collagen type I from mammalian sources,^[^ ^43^ ^]^ the characteristic collagen UV‐CD band at 220 nm started to vanish completely between 40 and 42 °C suggesting the successful unfolding of the collagen triple helix^[^ ^40^, ^41^, ^43^ ^]^ Cooling the ColMA back to room temperature resulted in a slight recovery of the bands which could be entirely recovered by keeping the sample for 2 days at 4 °C similar as it has been observed for native collagen.^[^ ^26^ ^]^

**Figure 3:**
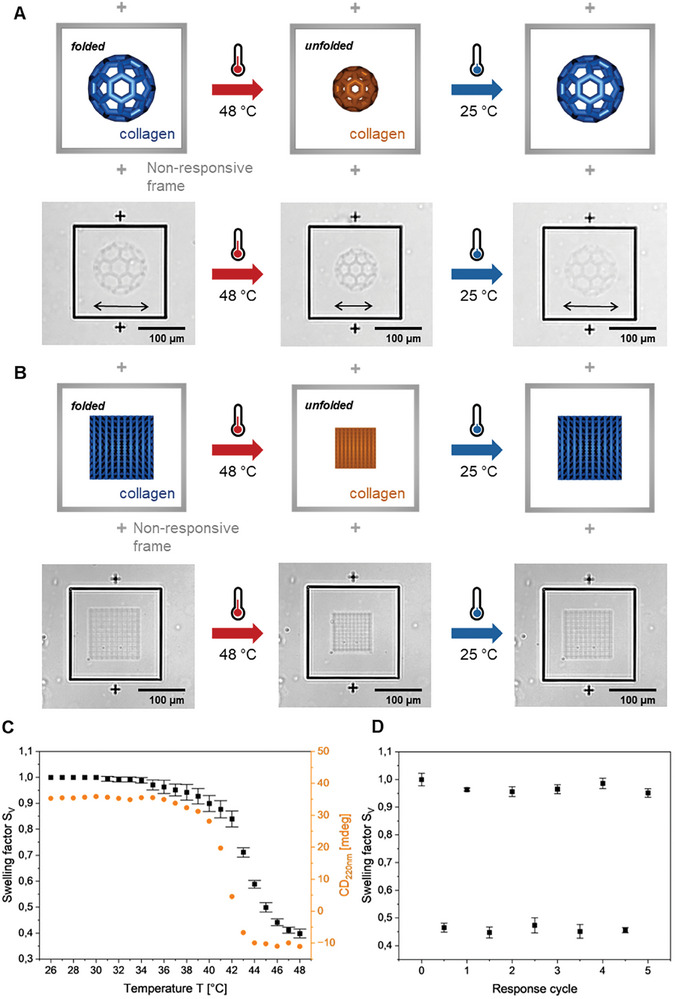
Analysis of the temperature response of 3D printed collagen microstructures. A,B): Temperature‐response of two‐photon 3D printed collagen buckyballs (diameter of ≈100 µm) and cubic lattice grids (100 µm × 100 µm × 70 µm) and a non‐responsive frame of commercial printed IP‐S (Nanoscribe GmbH). Models (up) and optical microscopy images (down). Upon heating, the microstructure shrinks due to collagen unfolding, and cooling the microstructures back to 25 °C entirely recovers the initially printed structure. Further images with multiple printed fields can be found in the Supporting Information (see Figure S26, Supporting Information). C) Volumetric swelling factor (S_V_) of 3D microprinted cubic lattices (100 µm × 100 µm × 70 µm) upon heating (black). UV‐CD signal at 220 nm of 5 µg mL^−1^ ColMA in 0.02 m acetic acid solution upon heating to different temperatures (orange). The change at 220 nm occurs due to the unfolding of collagen. D) Temperature response over multiple response cycles. The swelling factors are calculated from images at room temperature of cubic lattices before and after heating to 48 °C.

Next, we investigated if the reversible self‐assembly, i.e., unfolding and folding transition, also takes place in the 3D‐printed collagen microstructures. To this aim, we printed the previously optimized 3D microstructures consisting of buckyballs with larger dimensions (≈100 µm diameter) (see **Figure** **3**A). As a reference, frames of non‐responsive commercial material (IP‐S, Nanoscribe GmbH) were printed around. After two‐photon 3D laser printing, the 3D printed buckyballs were observed by optical microscopy varying the temperature from 25 to 48 °C (and vice versa) with 1 °C min^−1^ (Video S1, Supporting Information). Upon heating, the micrometric buckyballs shrank isotropically. Cooling the heated microstructures back to room temperature fully recovered the initial dimensions of the buckyballs. The response in the same temperature range indicates that the reversible temperature‐responsive unfolding also occurs in 3D‐printed (crosslinked) collagen.

Quantitative analysis of this reversible temperature response was performed with cubic lattice structures with dimensions of 100 µm × 100 µm × 70 µm (Figure 3B and Video S2, Supporting Information). In detail, the top layer areas of cubic lattice structures were measured and used for calculating the volumetric swelling factor (S_V_) with increasing temperature, respectively (Figure S26, Supporting Information). The onset point was observed at ≈41 °C, which is in good agreement with the transition temperature detected for the collagen unfolding by UV‐CD spectroscopy (see Figure 3C). A total volume reduction of over 50% (S_V_ = 0.45) was found upon heating. This behavior was found to be reversible over multiple times (5 cycles) exhibiting similar volume changes (S_V_ = 0.45‐0.50), i.e., shrinkage, upon heating and full recovery to the initial “swollen state” (S_V_ = 1.00‐0.95) upon cooling (Figure 3D). The highly reproducible response proves reversible self‐assembly and the absence of defects in the 3D‐printed collagen microstructures.

To investigate the effect of the printing parameters on the response, we have printed the cubic lattices with increasing scanning speed in 20 mm^−1^s^−1^ step from 20 to 100 mm^−1^s^−1^, which should result in decreasing crosslinking density. (see Figures S27,S28, Supporting Information). Although the shrinking was found to be similar, the recovery rate upon cooling to room temperature decreased with the reduction of different crosslinking densities. For lower crosslinked structures, full recovery was observed after 1 h at room temperature. Enhancing recoveries with higher crosslinking were previously reported for the recovery of shape memory polymers suggesting that the mechanism of folding can be described as similar to the recrystallization process.^[^ ^44^, ^45^, ^46^, ^47^ ^]^

**Figure 4:**
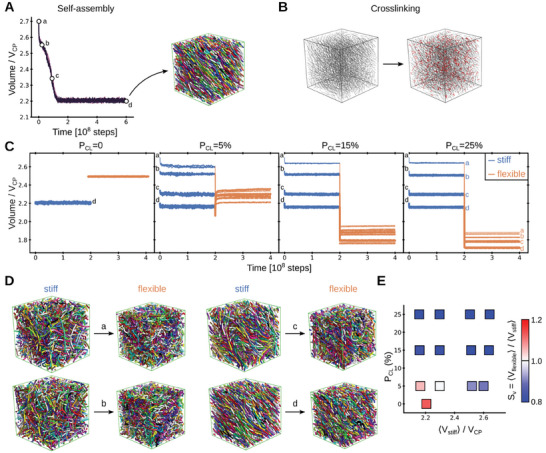
Reducing bending stiffness induces the shrinkage of crosslinked polymers in stochastic molecular dynamics simulations. A) Volume of the system during self‐assembly of a mixture of semiflexible polymers (*n* = 3, lines). The volume is normalized by the theoretical closed packing volume, V_CP_, and time is given in the integration steps. For each replica, four conformations with different volumes were selected (white circles, labeled a, b, c, d). The snapshot shows the last of such conformation (polymers shown as ribbons of different colors). B) Upon self‐assembly, polymers were crosslinked with crosslinking probability P_CL_, as exemplified here for one system (polymers in gray and crosslinks in red). C) Volume time traces are shown for a stage during which the crosslinked polymers were kept semi‐rigid (“stiff”: blue) and for a subsequent stage in which they were set fully flexible (“flexible”: orange). N = 3 replicas were conducted for each of the four distinct initially considered volumes (different lines and labels a–d) and different crosslinking probability, P_CL_ (panels). For comparison, time traces for P_CL_ = 0 (no crosslinks) are also shown (here only one volume is applicable, corresponding to “d” in A). D) The shrinking process is illustrated for the four cases indicated in C for P_CL_ = 25%. Polymers are shown as ribbons of different colors. E) The swelling factor, Sv = <V_flexible_>/<V_stiff_>, i.e., the ratio between the average volumes in the “flexible” and the “stiff” stages, respectively (<> denoting time average), was obtained as a function of the volume in the stiff stage (<V_stiff_>: x‐axis) and the crosslinking probability (P_CL_: y‐axis). S_V_ is depicted in color according to the color scale at the right. Accordingly, shrinking upon flexibilization of the polymers is shown in blue and swelling in red.

Stochastic molecular dynamics simulations were performed to support the molecular basis of the shrinkage of collagen microstructures by heating. For this purpose, we mimicked the microstructure as a mixture of semi‐flexible crosslinked polymers. We initially let the mixture self‐assemble under equilibrium conditions and chose conformations at four different stages during this process for further simulations (see **Figure** **4**A). Subsequently, polymer chains were crosslinked with different crosslinking probability, P_CL_ (Figure 4B). Crosslinking helped to maintain the volume of the system almost constant, not only for the final stable conformation, with the lowest volume but also for conformations with larger volumes which would be unstable without crosslinks (Figure 4C).

Heating, above the phase transition temperature, transitioned collagen from a folded conformation into a random coil in experiments (see Figure 3C; Figures S23–S25, Supporting Information). Folded collagen triple helices display persistence lengths varying from few to several tens of nm,^[^ ^48^, ^49^, ^50^ ^]^ meaning they are at least an order of magnitude stiffer than unfolded protein chains (for which persistence lengths are ≈1 nm).^[^ ^51^, ^52^, ^53^ ^]^ We modeled this effect by reducing the bending stiffness of the individual polymer chains (by ≈25‐fold in terms of their persistence length) while still maintaining the crosslinks between them (see Experimental Section). Accordingly, the system turned from a mixture of “stiff” into one of “flexible” crosslinked polymers (we note that our simplified coarse‐grained approach ignores other potential factors such as the change in topology when three chains form from one helix). Remarkably, this caused the volume of the systems to reduce in the majority of the crosslinked cases (compare “stiff” and “flexible” stages in Figure 4C and see representative conformational transitions in Figure 4D). The swelling factor was computed here as a function of two key free parameters: the volume before the coil transition (i.e., the volume in the “stiff” stage, <V_stiff_>) and the crosslinking probability (P_CL_) (Figure 4E). Substantial shrinkage was observed for crosslinking probabilities above 15%, independently of <V_stiff_>. Shrinkage was also observed for lower probabilities, but to a smaller extent and only provided that the initial volume was considerably high (<V_stiff_> > 2.4·V_CP_). In the absence of crosslinks, the system did not shrink but swelled instead (compare P_CL_ = 0 with P_CL_≠0 in Figures 4C,E). In summary, our simulations demonstrate that a mixture of cross‐linked polymers undergoes shrinking transitions, by loosening the bending rigidity of the constituent chains.

## 3 Conclusion

We have presented an easily accessible collagen ink suitable for two‐photon 3D laser printing, which can be stored at room temperature for several weeks and be used with commercial printing set‐ups, thereby significantly facilitating future applications. Furthermore, we have proved biocompatibility and preservation of fibrillar structure in the 3D printed collagen microstructures. Therefore, we believe that this approach paves the way for 3D laser printing of extracellular matrices to influence and support other biological studies on cell migration and tissue assemblies.^[^ ^54^, ^55^, ^56^ ^]^ Moreover, we have demonstrated for the first time the full reversibility of temperature‐induced self‐assembly, i.e., folding and unfolding, of collagen in 3D printed materials. Strikingly, full reversibility was found in multiple cycles leading to recurring large volume changes. This makes the new response mechanism particularly interesting for the future development of responsive biomaterials, with tunable changes in volume and molecular structure. For example, the temperature transition of the folding‐unfolding mechanism can be tuned either by varying the collagen source or by implementing synthetic collagen‐mimicking fragments.^[^ ^26^, ^57^, ^58^ ^]^ Thus, we also believe that these results provide an excellent platform for designing printed biomaterials that are responsive and whose properties can be modeled and predicted by theory.

## 4 Experimental Section

### Chemicals & Materials

Bovine collagen type I methacrylamide (ColMA) was used as purchased from Advanced BioMatrix with the product name PhotoCol. The photoinitiator lithium phenyl‐2,4,6‐trimethylbenzoylphosphinate (LAP) and HPLC grade water were purchased from Fisher Scientific. Acetic acid (0.1 m) was purchased from Merck and diluted with HPLC‐graded water to a concentration of 0.02 m. The fluorescent dye methacryloxyethyl thiocarbamoyl rhodamine B (RhodBMA) was purchased from Polysciences. All chemicals and samples were stored and handled under yellow light. SYLGARD 184 Silicone Elastomer Kit from Dow Chemical was used for the preparation of polydimethylsiloxane (PDMS) molds.

### Preparation of Collagen Ink

ColMA (12 mg) was first dissolved in 1.5 mL of acetic acid (0.02 m) by vigorously stirring the lyophilized solid for 3 h at 4 °C. Next, LAP (10.5 mg) was dissolved in 300 µL water and added to the dissolved ColMA solution. Last, RhodBMA (1 mg) was added to 1 mL of water and sonicated for 15 min in a sonicator bath to get a saturated aqueous RhodBMA solution. After filtration of the solution with a syringe filter (0.2 µm), 300 µL of the saturated dye solution was added to obtain the final collagen ink.

### Characterization of Collagen Ink

The pH was measured with METTLER TOLEDO S220 SevenCompact. The flow properties of the inks were measured by rotational rheometry using a HAAKE MARS rheometer with a measuring geometry of C60/1° Ti‐L. The shear rate varied between 100 and 1500 s^−1^ in 30 steps. Refraction indices were determined at 20 °C and the two‐photon laser printing wavelength 780 nm using a Schmidt+Haensch ATR L refractometer. UV–vis and FT–IR spectroscopy of the collagen ink was measured with a Jasco V‐770 and a Jasco FT/IR‐4600 spectrometer, respectively. UV–vis spectroscopy was performed using a Quartz cuvette (d = 2 mm). Before cryo‐electron microscopy, 3 µL of the collagen ink was applied to a glow discharged (3s, Solarus Gatan) Quantifoil (2/1, copper) specimen support grid and blotted in a humidified atmosphere for 4 s (Vitrobot, Thermofisher). The sample was vitrified by plunging it into liquid ethane. Grids were mounted under liquid nitrogen in autogrids and stored. The ink was imaged using similar conditions as the TEM sections (see Sectioning and imaging using electron microscopy in Experimental Section).

### Silanization Procedure

Glass coverslips (Marienfeld, 170 ± 5 µm) and indium tin oxide (ITO)‐coated glass coverslips (SPI Supplies, 160–190 µm, 70—100 Ω sq^−1^) were washed with isopropanol and acetone and dried with pressurized nitrogen. Subsequently, the surface was activated for 1 min by plasma treatment using a TDK PiezoBrush. The coverslips were immersed in a 4 mm solution of 3‐(trimethoxysilyl)propyl methacrylate in toluene for 1.5 h. The methacrylate‐functionalized coverslips were further washed twice in toluene and once in acetone and used as substrates for microprinting.

### Two‐Photon Laser Printing of 3D Collagen Microstructures

Two‐photon laser printing was performed with a commercially available set‐up (Photonic Professional GT2, Nanoscribe GmbH & Co. KG) in an oil immersion configuration and a femtosecond laser wavelength of 780 nm. All structures were printed using a 25× oil objective (NA = 0.8) from Zeiss for focusing the laser inside of the ink. Before printing, GWL files were generated with the Describe software (Nanoscribe) from previously designed STL files of desired geometries by setting slicing and hatching to 300 nm for all printed geometries, respectively. Before printing, a PDMS mold was prepared and placed on the methacrylate‐functionalized coverslips (22 mm × 22 mm) to avoid evaporation of solvent during the printing process. Next, collagen ink (5–30 µL) was applied to the PDMS mold before the filled mold was covered with a round glass slide from the other side. The sandwich cell was inserted into the Nanoscribe GT2 and printed with scanning speeds ranging from 20 to 60 mm^−1^s^−1^ and laser powers in the range of 21–42 mW depending on the structure and experiment. After micro printing, the round coverslip and residual collagen ink were removed, and the structures were washed 5 times with ≈100 µL of acetic acid (0.02 m) and 5 times with 100 µL of water always ensuring the structures never dried. Last, the PDMS mold was closed with a round glass slide again to avoid solvent evaporation. It is important to note that the structures need to be kept in aqueous media throughout the whole procedure and analysis to avoid irreversible collapse of the collagen network upon drying.

### Optical and Confocal Fluorescence Microscopy

Optical (fluorescence) microscopy images were recorded with a Zeiss Axio Imager M2 using a 5× long distance Zeiss objective (NA = 0.13) or an LD Plan‐Neofluar 20×/0.4 Korr Ph M27 objective (NA = 0.4) and an Axiocam 705 microscope camera. Fluorescence images were recorded with a Zeiss Axio Imager Z1 using an excitation wavelength of 555 nm. Fluorescent z‐stacks of collagen microstructures were recorded with a Nikon A1R confocal microscope equipped with GaAsP‐detectors using a 20× Nikon objective (NA = 0.8). A 559 nm diode laser was used for excitation, and the emission at 595 nm was collected. The structure was imaged in slices of 0.8 µm step width with a pinhole diameter of 1 au. Data was computed and analyzed using ImageJ. Cell viability assays were imaged using a Zeiss AxioObserver Z1 epifluorescence microscope, equipped with a heated incubation chamber and A‐Plan 5× objective (NA = 0.12) and LD A‐Plan 20× objective (NA = 0.35). Confocal fluorescence images of immunocytochemical staining were acquired with a Zeiss LSM800 confocal microscope equipped with a 40× oil immersion objective (NA = 1.4).

### Embedding Protocol for Electron Microscopy

Scanning and transmission electron microscopy images were acquired after embedding two‐photon 3D laser‐printed collagen structures in epoxide resin. For this purpose, 50 µm × 50 µm × 50 µm cubes were first printed on ITO‐coated glass coverslips and developed using the previously described development procedure with acetic acid (0.02 m) and HPLC water.

To avoid the collapse of collagen, a multi‐step embedding protocol was followed for successful fixation, heavy‐atom staining, dehydration, and critical point or embedding in an epoxide resin.

First, the structures were fixed by immersing them in 2.5% aqueous glutaraldehyde solution for 30 min. After washing the structures 3 times for 5 min with distilled water, the structures were immersed in a 3% aqueous tannic acid solution for 2 h at 4 °C. Removing the tannic acid solution and washing the printed structures 3 times for 5 min with distilled water was followed by staining and additional fixation with an aqueous solution of 2% OsO_4_ and 1.6% K_3_[Fe(CN)_6_] for 1 h at 4 °C. Washing the stained and fixed structures was performed twice for 5 min with distilled water before adding additional stain by immersing them in aqueous 2% uranyl acetate solution containing 25% ethanol at 4 °C overnight. On the next day, the stained and fixated structures were washed with a 25% ethanol‐water mixture twice for 5 min before being fully dehydrated in consecutive steps. This dehydration was performed by increasing the ethanol content from 25, 50, 70, and 90 to 100% in 10 min steps. Next, the ethanol was replaced stepwise by 50 and 100% acetone following the same time steps.

For critical point drying, acetone was replaced in 25 iterations at 17 °C by carbon dioxane using a Leica CPD300. For the embedding procedure, the solvent was replaced stepwise by mixtures of 30 and 70% epoxide resin in acetone, for 2 h each, followed by 100% epoxide for 1 h before being embedded in flat silicon molds. The molds were transferred into a heating oven and kept at 65 °C for 2 days. Removal of the glass substrate from the embedded sample was facilitated by placing the substrate in liquid nitrogen. Printing on ITO‐coated glass coverslips allowed easier separation of glass and embedding resin. It should be noted that 3D structures shrank to ≈70% of the original volume after fixation and embedding (see Figure S14, Supporting Information).

### Sectioning and Imaging using Electron Microscopy

Resin blocks were trimmed and sectioned using a Powertome PC ultramicrotome (RMC Boeckeler, Tucson, USA) equipped with diamond knives (Diatome, Biel, Switzerland) for trimming (Trim20) and cutting (Ultra 35). For SEM imaging (Ultra 55, Carl ZEISS Microscopy, Oberkochen, Germany) 70 nm sections were collected on pieces of Si wafer and post‐stained with 3% aqueous uranyl acetate followed by 3% aqueous lead citrate (Science Services, München, Germany). Hierarchical imaging at 1.5 keV landing energy using SE and ESB detectors was performed with the Atlas 5 software package (Carl ZEISS Microscopy). For TEM imaging 60 nm sections were collected on 200 mesh Quantifoil 2.2 Au grids (Quantifoil, Jena, Germany) and post‐stained with 3% uranyl acetate for 1 min.

For TEM images of the printed material, 70 µm sections of the embedded and stained material were placed on a QF grid and observed in a Krios (Thermofisher) electron microscope at 300 kV, equipped with an autoloader. Images were taken under cryo and low dose conditions at a pixel size of 2.8 Å with a Gatan K3 camera operated in counting mode. At an underfocus ≈2 µm movies were collected with a total dose of ≈20 e/Å2 using EPU software (Thermo Fischer) and corrected for beam‐induced motion (Motioncorr2 in Relion).

### Cell Viability Assay

Rat embryonic fibroblast cells were seeded onto collagen scaffolds, fabricated by two‐photon 3D laser printing, and cultivated in DMEM supplemented with 10% FCS from Pan‐Biotech (Aidenbach, Germany) in a humidified incubator at 37 °C and 5% CO_2_ overnight. Cell viability was verified using the LIVE/DEAD Viability/Cytotoxicity Kit from Invitrogen (Waltham, MA, USA), which applies calcein‐AM and ethidium homodimer‐1 (EthD1) to label live and dead cells, respectively. In addition, cell nuclei were stained with Hoechst 33342 from Invitrogen (Waltham, MA, USA). Labeling solutions were allowed to incubate for 30 min at 37 °C. Cells were imaged in PBS containing calcium and magnesium from Pan‐Biotech (Aidenbach, Germany) using epifluorescence microscopy and a 5× or 20× objective lens. The excitation wavelengths were 401, 493, and 577 nm, respectively.

Live and dead cells were characterized using Fiji. First, a mask was created from the cell nuclei based on the Hoechst labeling. For live cells, a threshold was applied to the calcein‐AM labeling to binarize the image. The binarized image was analyzed using the “analyze particles” plugin, restricting the particle size to 15‐infinite µm. Detected particles, which did not correspond to a nucleus, were excluded from analysis. For dead cells, a threshold was applied to the EthD1‐staining to cut off the fluorescence of the RhodBMA‐labeled collagen. The previously created mask was applied to analyze the nuclei for EthD1‐labeling using the “analyze particles” plugin, restricting the particle size to 5‐infinite µm. If multiple particles were detected per nucleus, they were counted as one. Percentiles of live and dead cells were calculated using Microsoft Excel.

### Immunocytochemical Staining

Immunostaining was performed to visualize the cell nuclei, focal adhesions, and actin cytoskeleton. Cells were fixed 24 h after seeding in 5% paraformaldehyde and permeabilized with PBS containing 0.1% Triton‐X100. The samples were incubated with 5 µg mL^−1^ anti‐Vinculin antibody (Invitrogen (Waltham, MA, USA)) in 1% (w/w) bovine serum albumin in PBS, followed by washing with PBS with 0.1% Triton‐X100 and incubation with secondary antibody donkey anti‐mouse Alexa Fluor 647 (Invitrogen (Waltham, MA, USA)), DAPI (10 µg mL^−1^) and Alexa Fluor 488 Phalloidin (1.5 U mL^−1^). Staining solutions were allowed to incubate for 1 h at room temperature in a humidity chamber.

### Temperature‐Responsive Reversible Folding and Unfolding Behavior

Heating of microprinted collagen was performed with a heating stage (LTS 420, Linkam Scientific Instruments) in the optical microscope. The transmission illumination mode with differential interference contrast and reflection mode with circular differential interference contrast were used for imaging. The investigated temperature programs started at 25 °C and heated the printed collagen to 48 °C using a gradient of 1 °C min^−1^. The heated microstructures were subsequently cooled using a gradient of 1 °C min^−1^ back to room temperature. The swelling factor S_V_ was calculated by estimating the volume from the top area of the printed cubic lattice grids. The top layer area of five lattices was used as an average.

UV‐CD spectra of the ColMA were recorded with a Jasco J‐1700‐CD spectrometer between 190 and 260 nm with a scanning speed of 100 nm min^−1^. To detect the CD signal, ColMA was diluted with 20 mm acetic acid to a concentration of 5 µg mL^−1^ or below for the measurement. Heating of the ink was performed at a speed of 1 °C min^−1^ while measuring the temperature of the holder. After cooling the cuvette and recording a CD spectrum at 4 °C, the cuvette was stored for 2 days at 4 °C before remeasuring the CD signal.

### Stochastic Molecular Dynamics Simulations

We modeled a fraction of the 3D‐printed collagen microstructures as a mixture of semiflexible crosslinked polymers. The mixture was constituted of 200 chains, each one with a degree of polymerization of 50. Each chain was modeled as a series of beads (one per monomer) connected by harmonic springs, with a bonded interaction energy V_bond_ = K_bond_(d‐2r)^2^/2. Here, K_bond_ is the elastic constant, d is the distance between consecutive beads, and 2r is the equilibrium distance. Pairs of beads i and j sterically repelled from each other, via consideration of the repulsive term of a Lennard‐Jones potential V_rep_ = 4ε(σ/d_ij_).^12^ Here, ε is the strength of the potential, σ relates to the radius of each bead (i.e., each monomer) via r = σ(2^1/6^)/2, and d_ij_ corresponds to the separation between beads. The repulsive potential was considered for all beads within a cutoff distance (d_cutoff_) and it was excluded for neighbor bonded beads. The bending rigidity of the chains was imposed by introducing an angular potential between all triads of consecutive bonded beads of the form V_angle_ = K_θ_[cos(θ)‐cos(θ_0_)]^2^/2, where K_θ_ is the strength of the potential, θ is the angle formed by the triad of beads and θ_0_ = 180°. Stochastic molecular dynamics were carried out to monitor the dynamics of the mixtures using the GROMACS MD package (version 2020.3) (“sd” integrator option).^[^ ^59^ ^]^ For each bead, the friction coefficient was assumed to be mγ, where m is the bead mass and 1/γ is the relaxation time. The friction coefficient is related to the diffusion coefficient, D, via D = k_B_T/mγ, where k_B_ is the Boltzmann constant and T the temperature. Langevin equations of motion were numerically solved at discrete time steps Δt. Neighbors were treated according to the Verlet buffer scheme.^[^ ^60^ ^]^ The length was normalized by the separation between beads (related to) σ, time by the characteristic diffusion time of a bead τ = (2r)^2^/D, mass by the mass of a bead, and energy by k_B_T (see list of simulation parameters in Table S2, Supporting Information).

The chains were assumed to adopt initial linear conformations and were placed at random positions and orientations, without overlap, in a cubic box of dimensions (399.6 σ).^3^ The box dimensions were reduced gradually in two consecutive steps, first, during 10^7^ integration steps until the system reached a volume of (48 σ)^3^ and second, during 10^6^ integration steps until the volume was (30 σ).^3^ From this point on, the system was simulated under equilibrium NPT conditions. The system was coupled to a Berendsen barostat to maintain the pressure constant at 1 bar (coupling time of 5 in GROMACS arbitrary units equivalent to ≈0.07937 τ).^[^ ^61^ ^]^ In addition, Langevin dynamics ensured the temperature stayed constant at 300 K (see resulting k_B_T in Table S2, Supporting Information). In the first stage, the system was allowed to further self‐assemble for 6·10^8^ integration steps (simulation length ≈0.952·10^6^ τ). N = 3 simulation replicas were conducted. Four configurations with different volumes were extracted from each replica for further simulations (times indicated with the white circles in Figure 4). In the second stage, crosslinks between chains were added. For the four selected configurations, pairs of monomers that belonged to different chains, but which were in close proximity (at a distance smaller than 2r) were connected by a harmonic spring with a crosslinking probability P_CL_ = 5, 15, or 25%. The elastic constant of the spring was set to K_bond_ and the equilibrium reference distance to 2r. Subsequently, the dynamics of the resulting crosslinked mixture were simulated during 2·10^8^ integration steps (simulation length ≈0.317·10^6^ τ). This stage was denoted as the “stiff” stage in Figure 4. In the third stage, the bending rigidity of the chains was removed by setting the angular interaction potential V_angle_ = 0. The crosslinked (now flexible) mixture was simulated during 2·10^8^ integration steps (simulation length ≈0.317·10^6^ τ). Note that crosslinks between chains were maintained during this stage. This stage was denoted as the “flexible” stage in Figure 4. The persistence length of a single chain in isolation was predicted to be ≈50r, in the “stiff” stage, and 2r, in the “flexible” stage (i.e., a ≈25‐fold decrease upon release of the angular constraints). For comparison, simulations without crosslinks (P_CL_ = 0) were also carried out. In this case, the last 2·10^8^ integration steps of the self‐assembly process, for which the system already equilibrated (see Figure 4A), were considered as the “stiff” stage. For the “flexible” stage, additional simulations were conducted removing the angular restraints, starting from the final configuration upon self‐assembly.

The volume of the system was monitored throughout the different stages. It was normalized by the theoretical closed packing volume, V_CP_ = (32/π)·V_mon_·N≈1.35·V_mon_·N, for N = 10 000 monomers, each one with a volume V_mon_ = 4πr^3^/3. The swelling factor was computed as Sv = <V_flexible_>/<V_stiff_>, with <V_flexible_> and <V_stiff_> being the average volumes during the “flexible” and the “stiff” stages, respectively (<> denotes time average over the last 10^8^ steps of each stage combining the *n* = 3 replicas).

## Conflict of Interest

The authors declare no conflict of interest.

## Supporting information



Supporting Information

Supplemental Video 1

Supplemental Video 2

## Data Availability

The data that support the findings of this study are openly available in heiDATA, the Open Research Data institutional repository for Heidelberg University at https://doi.org/10.11588/data/WTFEHF.
