# Supplementary material for: Responsive 3D Printed Microstructures Based on Collagen Folding and Unfolding
Source: Small. 2024 Nov 27;21(3):2408597. doi: 10.1002/smll.202408597 (PMC11753499; doi:10.1002/smll.202408597)
Supplement: Supplementary file 1 — Supporting Information [file SMLL-21-2408597-s002.docx]

Supporting Information

**Responsive 3D printed microstructures based on collagen folding and unfolding**

Philipp Mainik ^1,2^, Camilo Aponte-Santamaría ^3^, Magdalena Fladung ^4^, Ronald Ernest Curticean ^5^, Irene Wacker ^5^, Götz Hofhaus ^5^, Martin Bastmeyer ^4,6^, Rasmus R. Schröder ^5^, Frauke Gräter ^3,7^, Eva Blasco ^1,2^*

*corresponding author (eva.blasco@uni-heidelberg.de)

*Affiliations*

^1^ Institute for Molecular Systems Engineering and Advanced Materials (IMSEAM), Heidelberg University, 69120 Heidelberg, Germany.

^2^ Organic Chemistry Institute (OCI), Heidelberg University, 69120 Heidelberg, Germany.

^3^ Heidelberg Institute for Theoretical Studies (HITS), 69118 Heidelberg, Germany.

^4^ Cell and Neurobiology, Zoological Institute, Karlsruhe Institute of Technology (KIT), 76131 Karlsruhe, Germany.

^5^ BioQuant, Heidelberg University, 69120 Heidelberg, Germany.

^6^ Institute for Biological and Chemical Systems - Biological Information Processing (IBCS-BIP), Karlsruhe Institute of Technology (KIT), 76344 Karlsruhe, Germany.

^7^ Interdisciplinary Center for Scientific Computing (IWR), Heidelberg University, 69120 Heidelberg, Germany.

Supporting Information

Outline

1. Characterization of collagen-based ink page S3

2. 3D microprinting of collagen and material characterization page S7

3. Temperature responsive of collagen in solution and in 3D printed structures page S14

**1. Characterization of collagen-based ink**


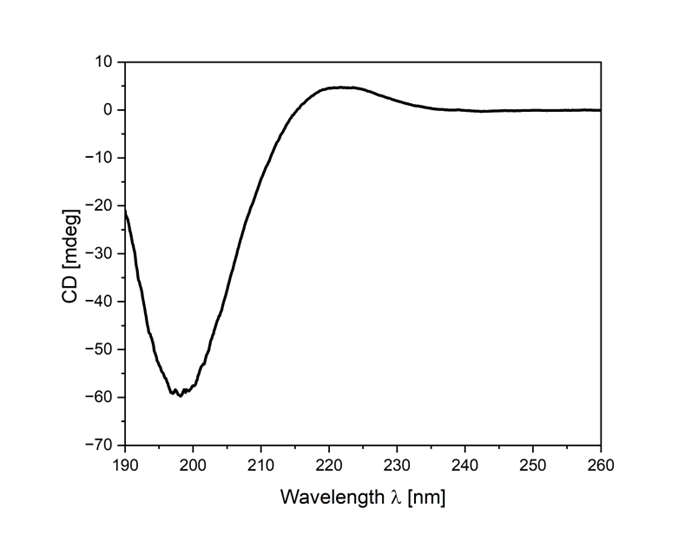


**Figure S1:** UV-CD spectrum of ColMA (2 µg/mL) in 0.02 M acetic acid aqueous solution at 25 °C.


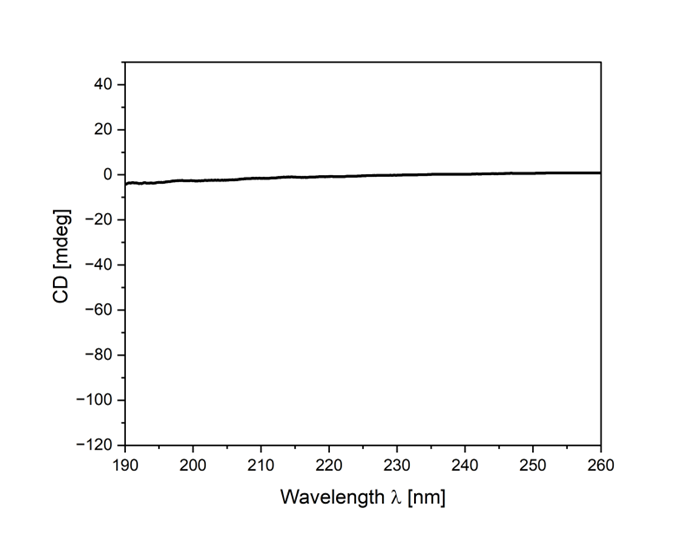


**Figure S2:** Reference UV-CD spectrum of 0.02 M acetic acid aqueous solution at 25 °C.


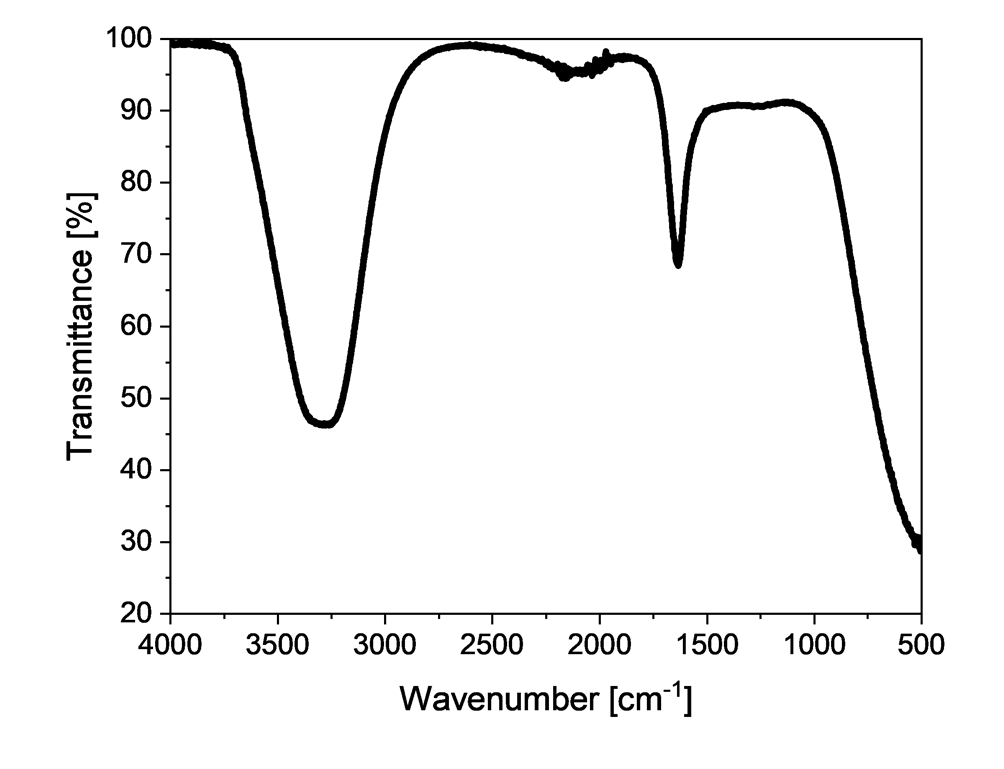


**Figure S3:** IR spectrum of the collagen methacrylamide ink showing two main absorption bands. The band at 3300 cm^-1^ corresponds to the O-H vibrational band of water. The band at 1600 cm^-1^ is in the region C=O vibration of the polypeptide backbone.


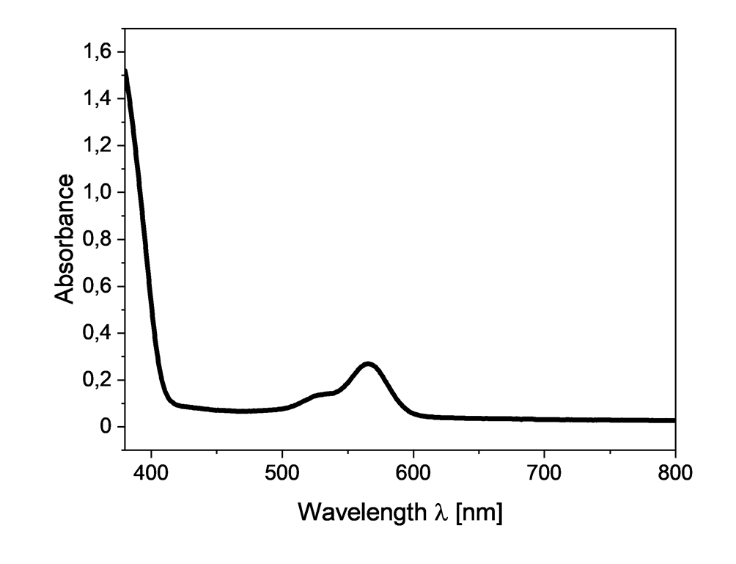


**Figure S4:** UV-vis spectrum of the collagen ink in a quartz cuvette with d = 2 mm showing strong absorption of the photoinitiator LAP below 400 nm. The two absorption bands between 500 nm and 600 nm correspond to rhodamine B methacrylate. Importantly, no additional absorption in the printing region at 780 nm was observed.


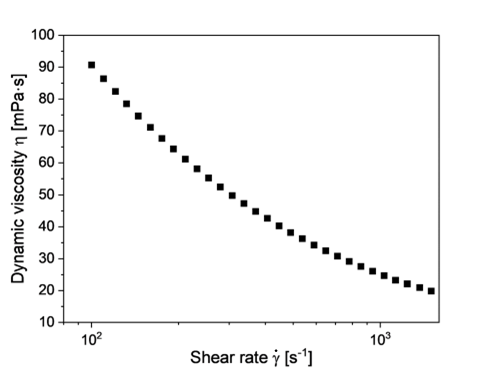


**Figure S5:** Measured dynamic viscosity at 25 °C for varying shear rates from 100 s^-1^ to 1500 s^-1^ using rotational rheology.


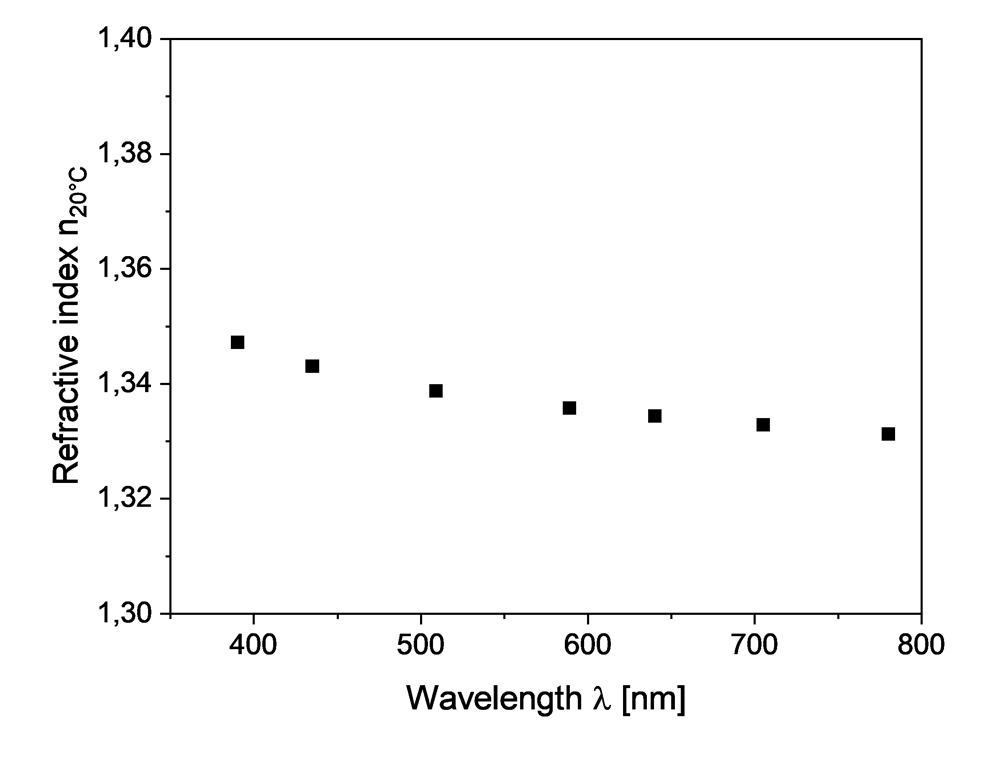


**Figure S6:** Measured refractive indices at 20 °C between 380 nm and 780 nm.


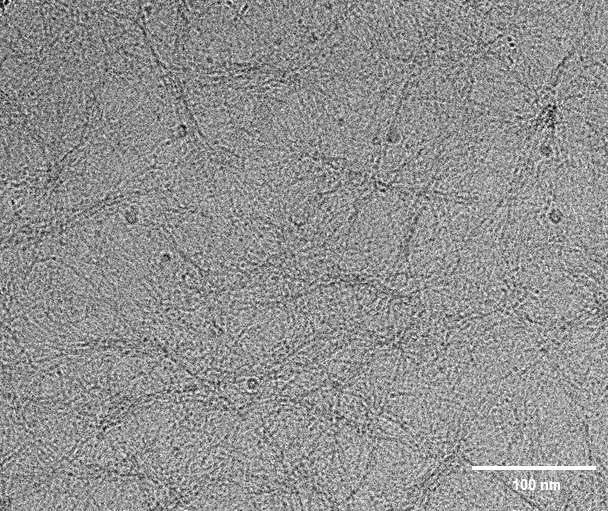


**Figure S7:** Cryo-EM imaging of collagen ink showing the collagen fibrils of around 13 nm diameter. Fibrils are visible due to the (phase) contrast of the protein versus water.


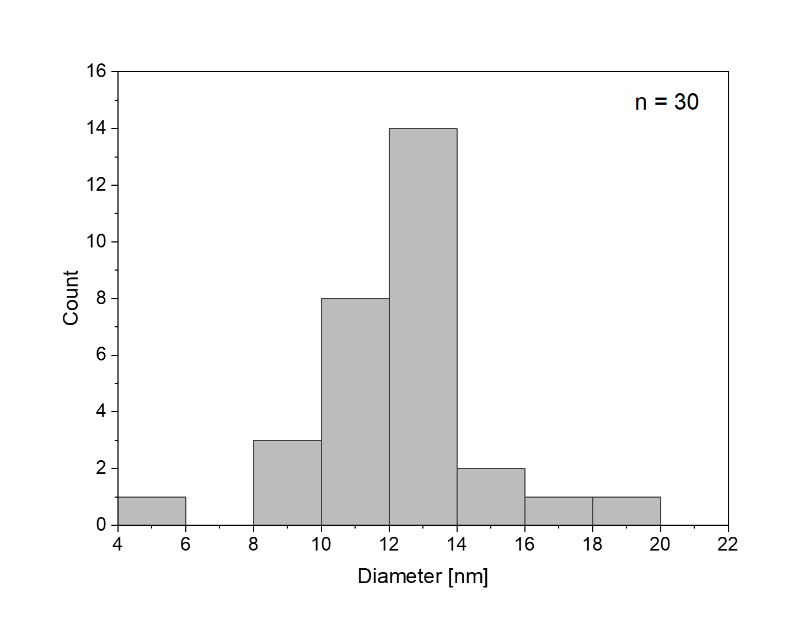


**Figure S8:** Histogram of n = 30 measured fibril diameters in the cryo-EM image. The average of measured diameters was calculated to 12.3 nm ± 2.5 nm.

**2. 3D microprinting of collagen and material characterization**


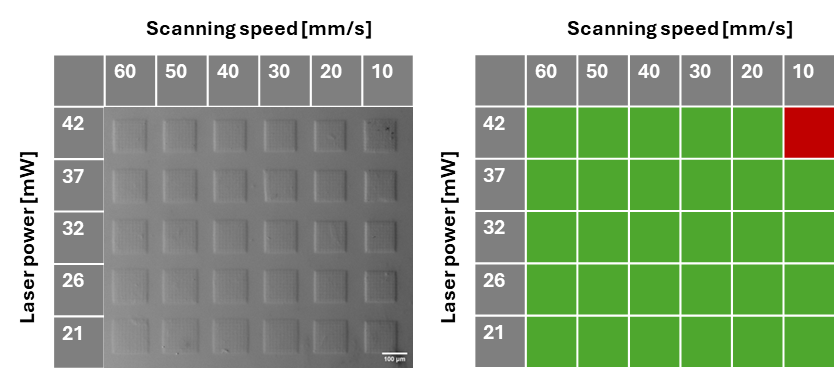


**Figure S9:** Printability window of collagen ink for various combinations of laser powers and scanning speeds for printing cubic lattices of 100 µm × 100 µm × 70 µm dimension. Green and red color coding correspond to printing with stable and overexposure parameters, respectively. Scale bar: 100 µm.


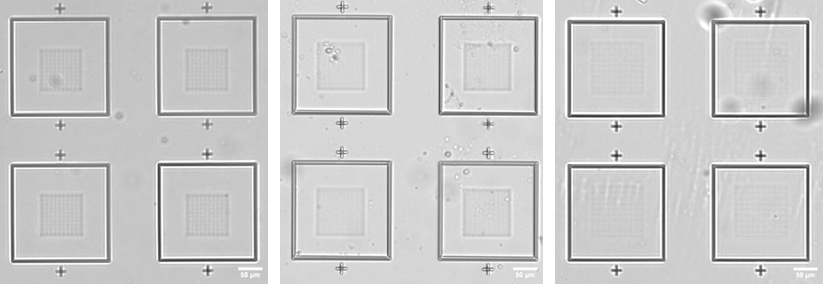


**Figure S10:** Stability of the printed collagen microstructures in various pH conditions (from left to right 2.56, 7.00, 10.82). To enhance the visibility of the crosslinked collagen methacrylamide, the hydrogels were printed inside of a frame of a commercial material (IP-S, Nanoscribe GmbH).


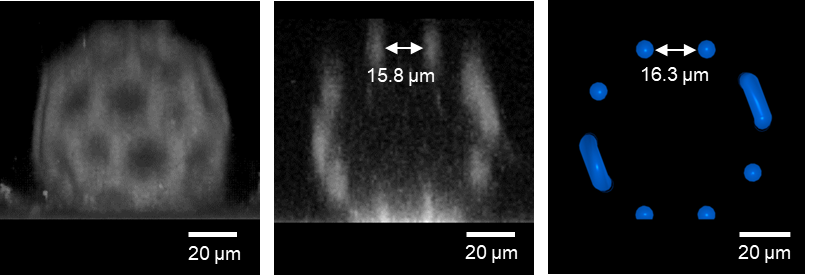


**Figure S11:** Side view on volumetric 3D reconstruction (left), vertical cross section (center) through the center of the printed buckyball structure (75 µm × 75 µm × 70 µm, 42 mW laser power, 20 mm/s scanning speed) after confocal fluorescence microscopy, and model of cross-section (right).


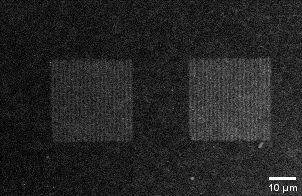


**Figure S12:** Resolution tests with parallel printed lines of 30 µm length with 1.5 µm spacing in-between. The printing parameters were 40 mW and 3 mm/s.


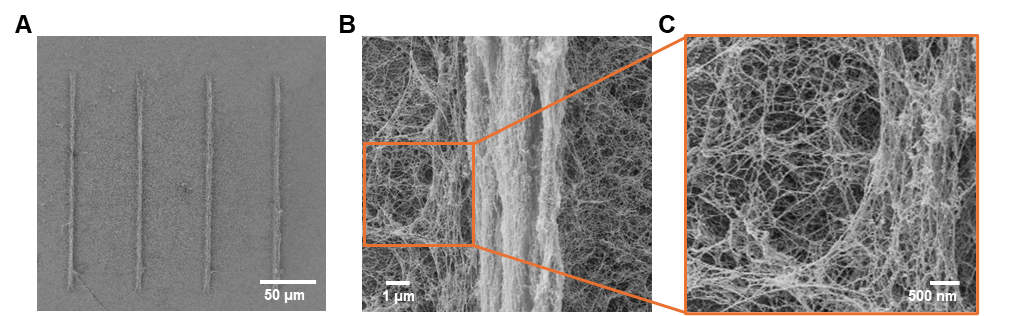


**Figure S13:** SEM images of critical point dried 3D printed collagen microstructures. The printed bars (A) with original dimensions of 200 µm × 10 µm × 10 µm collapsed under these preparation conditions to about 30% in width (B) and show an irregular fibrillar network (C).


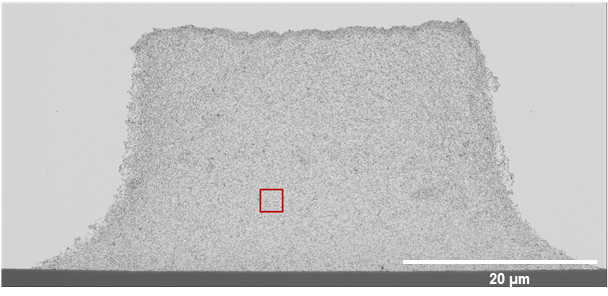


**Figure S14:** SEM image of cross section of epoxide embedded printed collagen. Shrinkage of the initially printed blocks (50 µm × 50 µm × 50 µm) to around 70% in each dimension occurred during staining and fixation with tannic acid and OsO_4_. Red square indicates the region used for analysis of fibril diameters.


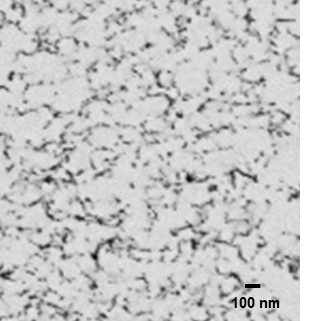


**Figure S15:** Zoom in SEM image of cross section of epoxide embedded printed collagen showing collagen fibrils of around 13 nm diameter. Note that SEM imaging at the low primary energy (1.5 keV) used here produces signal only from a thin surface layer of the section leading to seeming breaks or discontinuities in the collagen network.


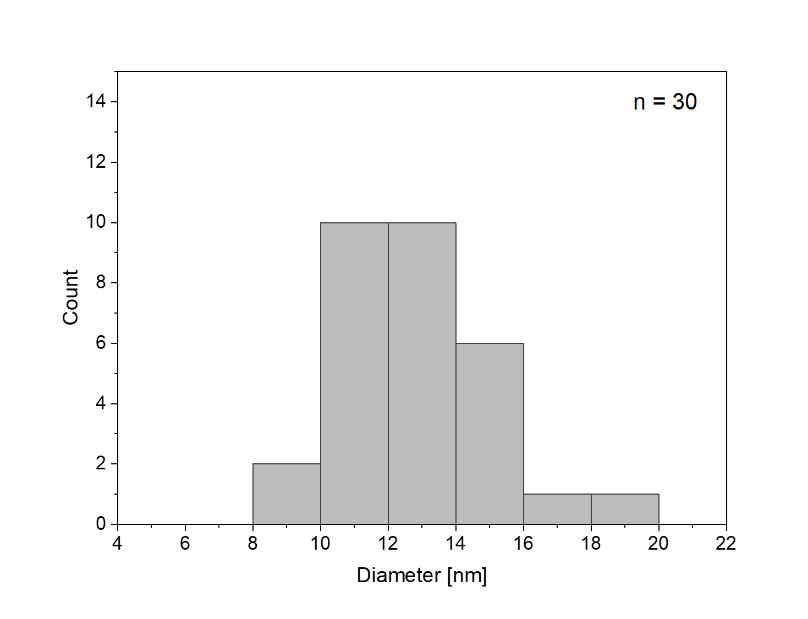


**Figure S16:** Histogram of n = 30 measured fibril diameters in the SEM cross section image. The average of measured diameters was calculated to 13.2 nm ± 2.4 nm.


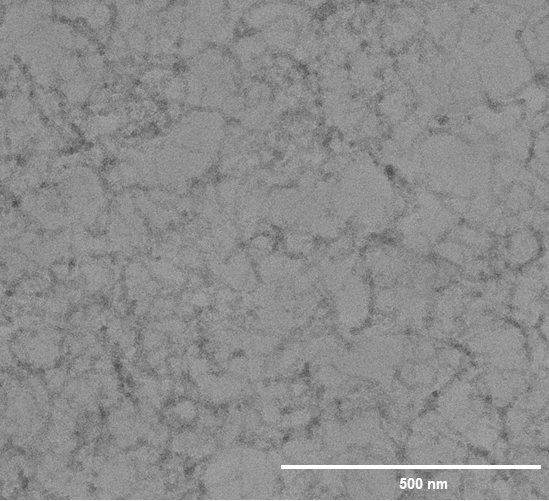


**Figure S17:** TEM image of cross section of epoxide embedded printed collagen showing collagen fibrils of around 13 nm diameter. Since in TEM a projection image is recorded a homogeneous fibrous network without discontinuities is visualized. Contrast in this image is created by the metals used to stain the embedded sample.


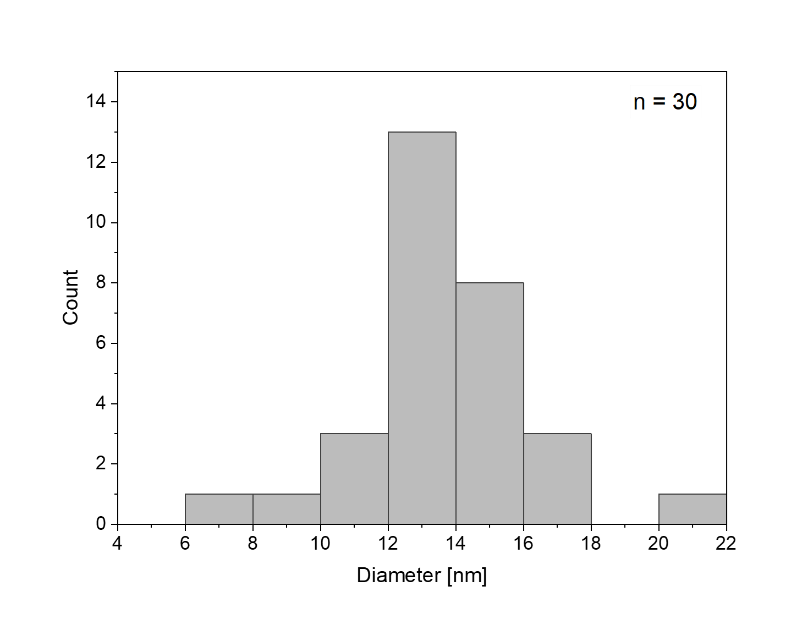


**Figure S18:** Histogram of n = 30 measured fibril diameters in the TEM cross section image. The average of measured diameters was calculated to 12.6 nm ± 2.3 nm.

**Table S1:** Fibril diameters obtained from different electron microscopy techniques for N = 30 averaged fibrils.

|  | Cryo-TEM (ink) | SEM (printed) | TEM (printed) |
| --- | --- | --- | --- |
| treatment | vitrified | stained + embedded | stained + embedded |
| sample | thinned liquid film | ultrathin section | ultrathin section |
| electron energy | 300 keV | 1.5 keV | 300 keV |
| fibril diameter | 12.3 nm ± 2.5 nm | 13.2 nm ± 2.4 nm | 12.6 nm ± 2.3 nm |


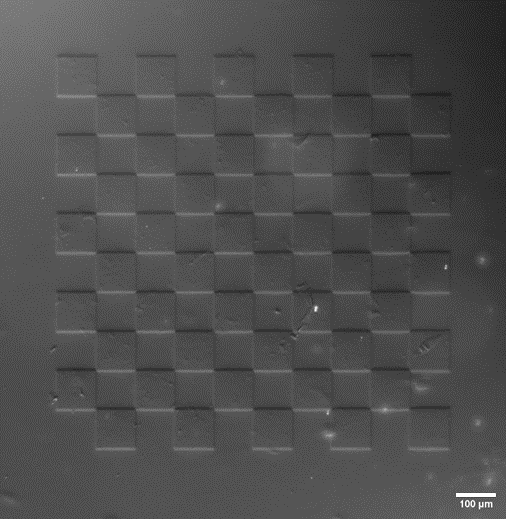


**Figure S19:** Optical microscopy image (acquired with CDIC contrast) of printed check board structure of collagen blocks for biocompatibility tests. Scale bar: 100 µm.


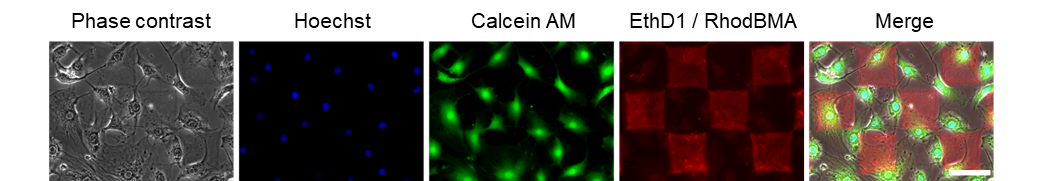


**Figure S20:** Cell viability assay REF cells on 2PPL printed collagen blocks. Cell nuclei are labelled with Hoechst (blue), live cells with Calcein AM (green) and dead cells labelled with EthD-1 (red). The RhodBMA-labelled collagen appears in the same color as the dead-stain (red). Within three independent experiments, the majority of cells did not appear positive for EthD1 labelling. Scale bar: 100 µm.


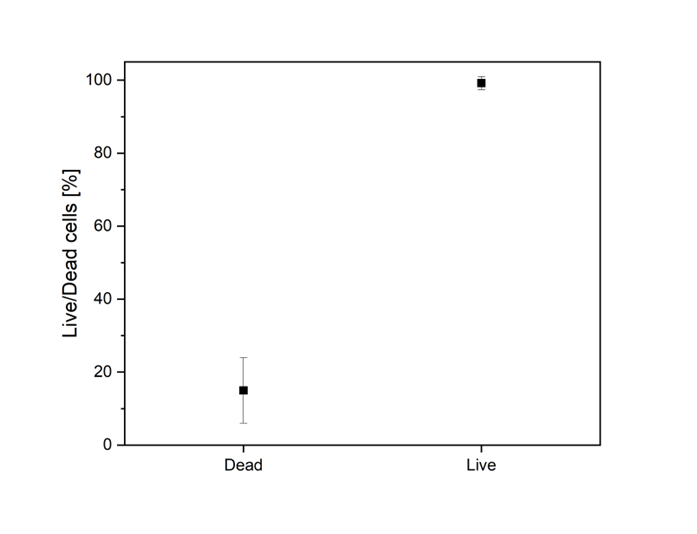


**Figure S21:** Results from statistical analysis of live/dead assay verifying >99% of cells to be alive. A fraction of 15% for dead cells can be traced back to the autofluorescence of the RhodBMA-labeled collagen. The total number of detected cell nuclei was N = 473.


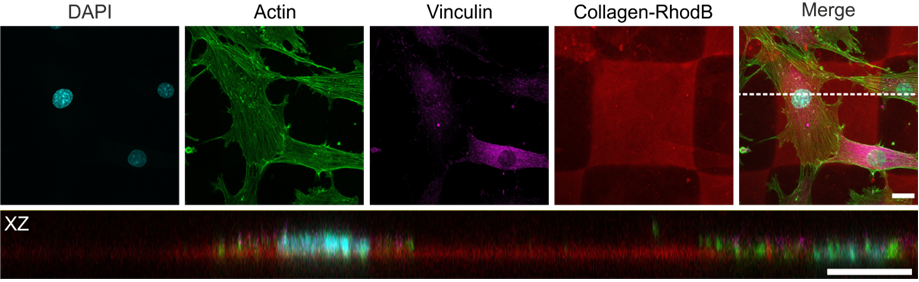


**Figure S22:** Confocal images of immunocytochemical staining of REF cells cultured on 3D laser printed collagen blocks. Cells adhered to the printed scaffold (red) and exhibit an undisturbed actin organization (green). Focal adhesions, as visualized by Vinculin-staining (magenta), formed on the RhodBMA-labelled collagen blocks (red). Cells adhered to the top of the scaffolds as shown by the XZ-projection. The projection plane is indicated in white in the merge image. Scale bars: 20 µm.

**3. Temperature response of collagen in solution and in 3D printed structures**


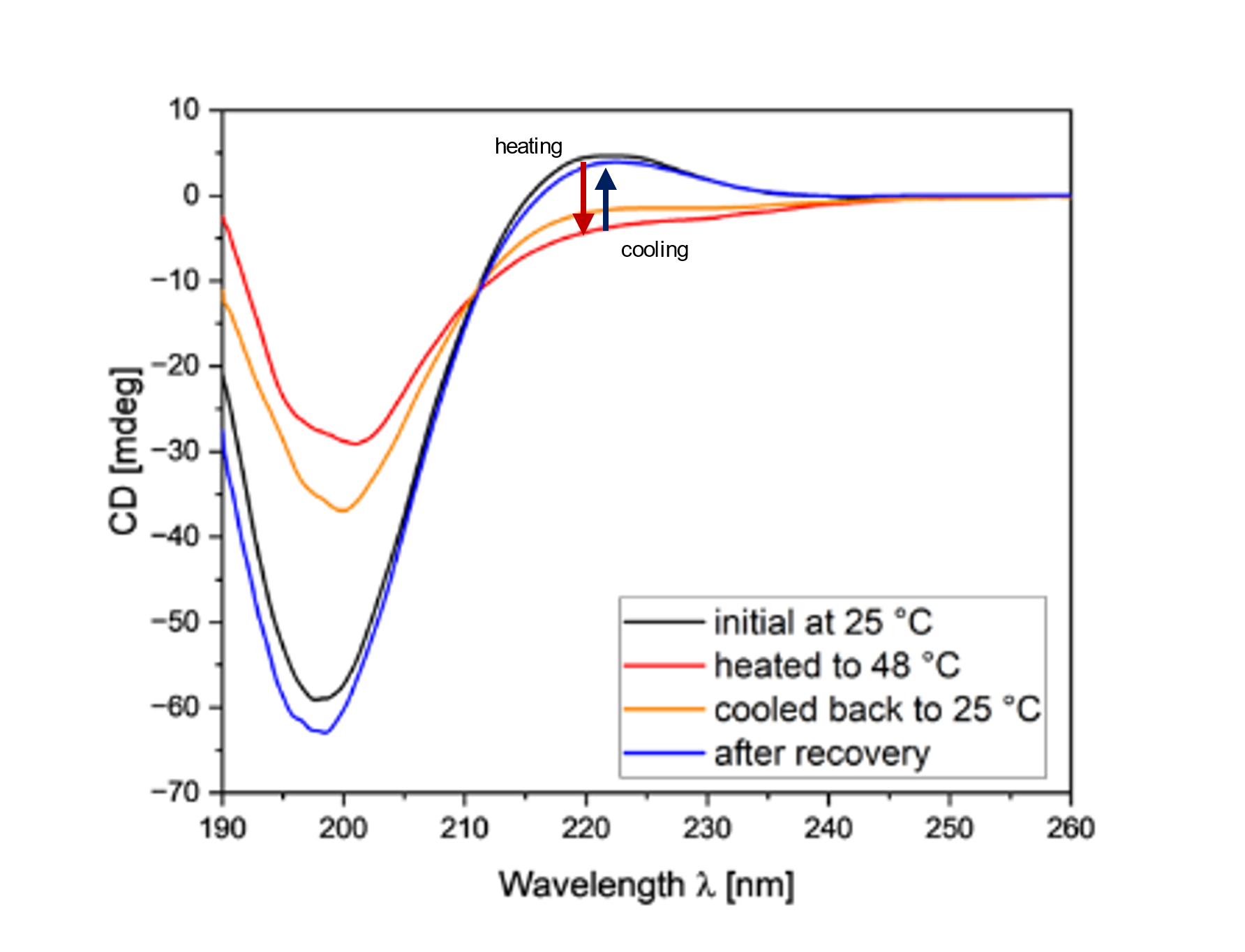


**Figure S23:** UV-CD spectra of 2 µg/mL ColMA in acetic acid solution (0.02 M) upon heating and cooling. Cooling of ColMA solution for 2 days shows full recovery of collagen folding bands at 200 nm and 220 nm which first disappeared upon heating.


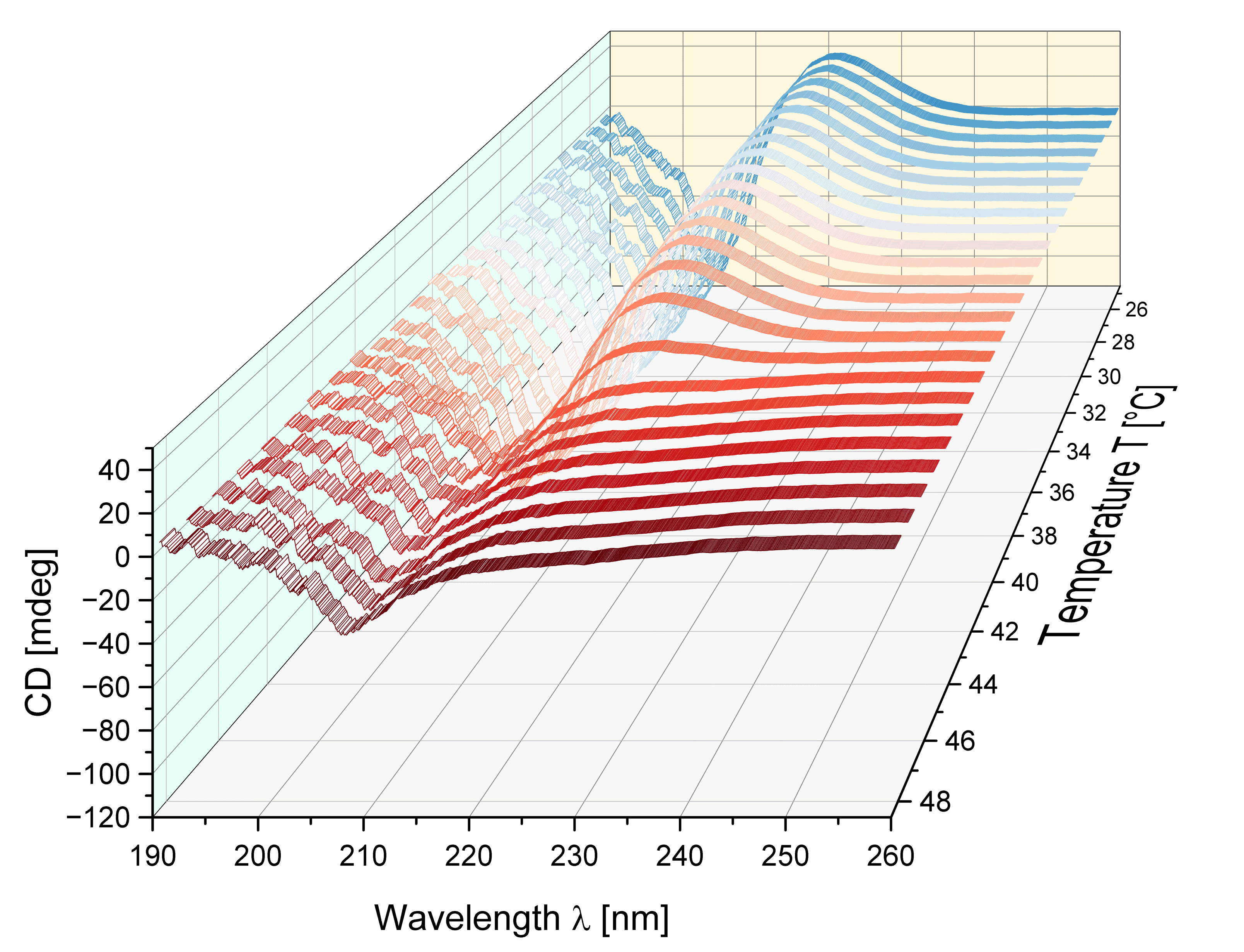


**Figure S24:** UV-CD spectra of 5 µg/mL ColMA in 0.02 M acetic acid solution at different temperatures.


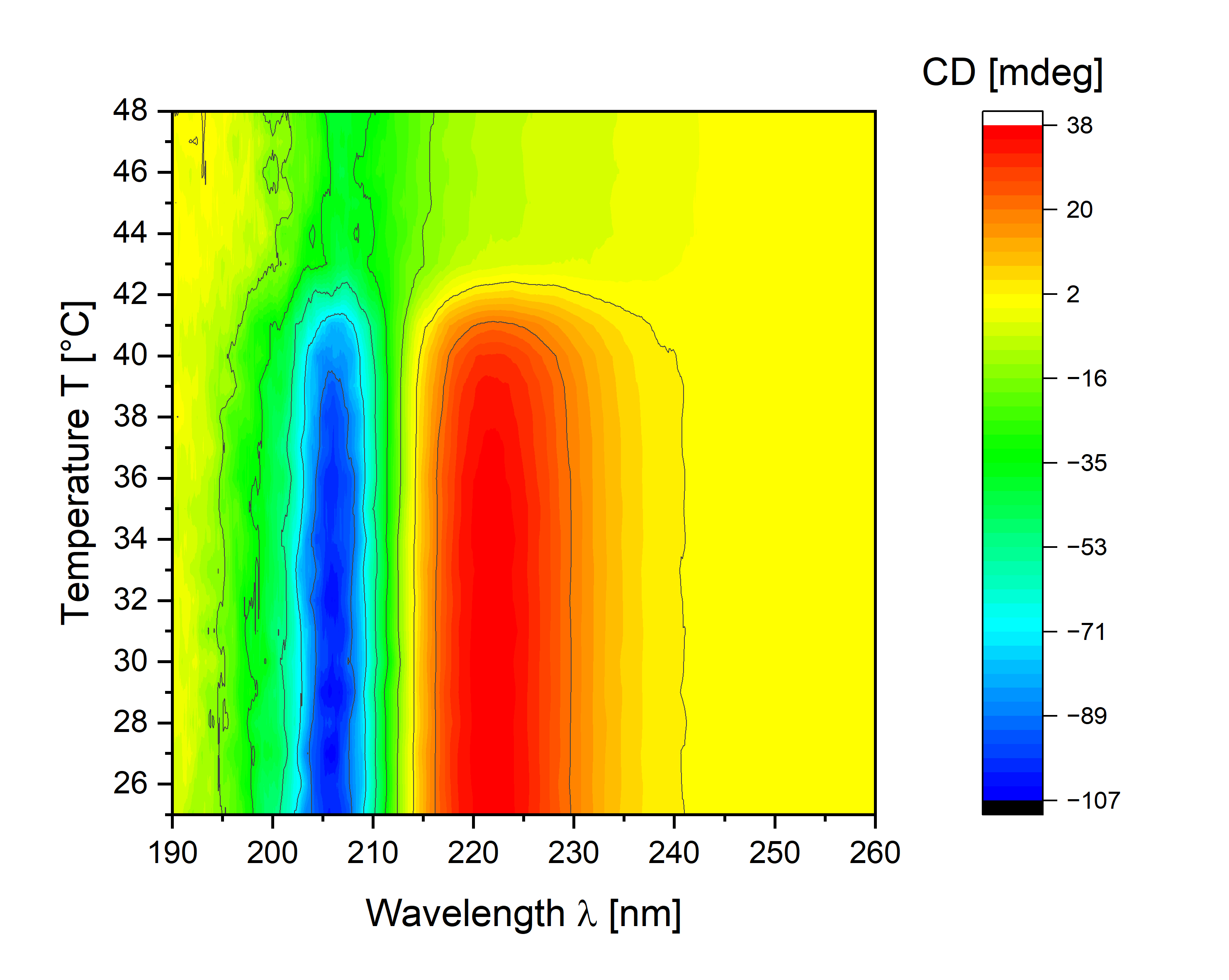


**Figure S25:** Contour plot of UV-CD spectra of 5 µg/mL ColMA in 0.02 M acetic acid solution at different temperatures.


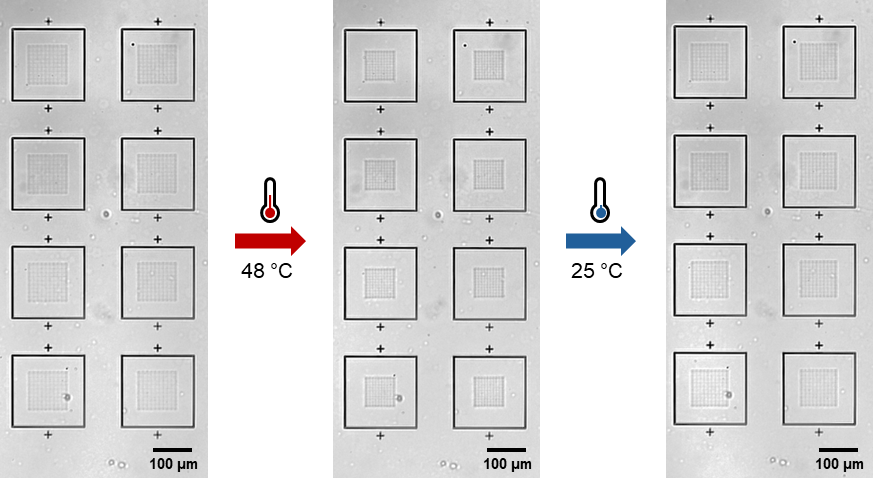


**Figure S26:** Response and recovery of printed collagen cubic lattices (100 µm × 100 µm × 70 µm) in printed frames of a non-responsive, passive material (IP-S, Nanoscribe GmbH) upon heating and cooling the sample by 1 °C/min.


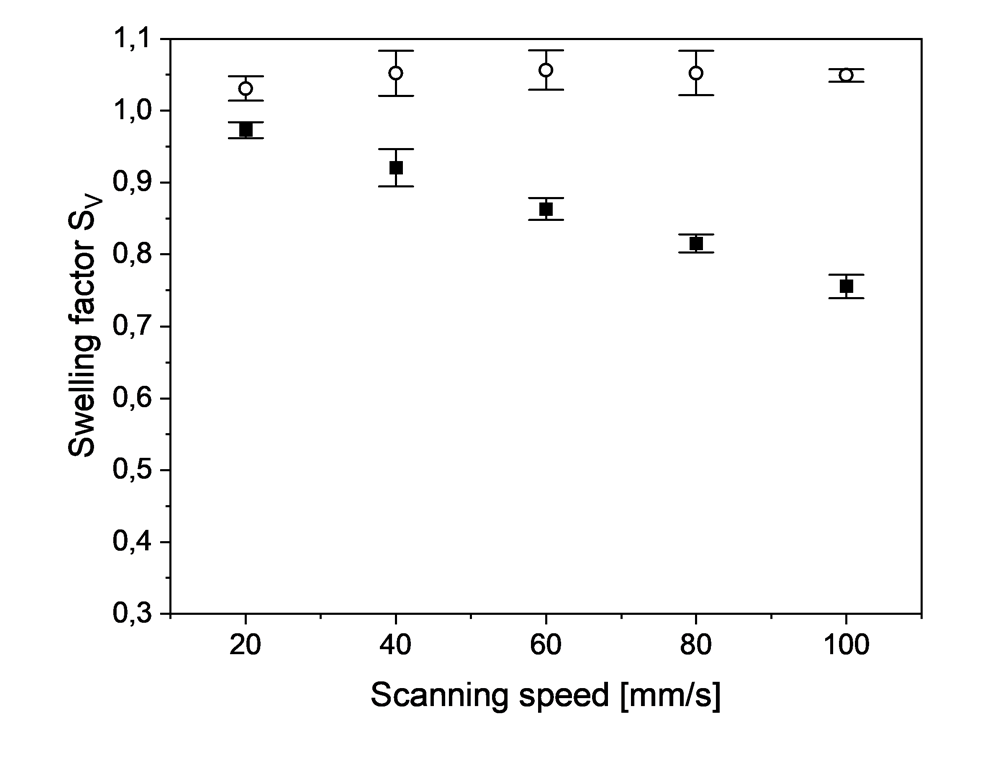


**Figure S27:** Recovery of collagen microstructures with 40 mW laser power and varying scanning speed (filled squares – recovery after cooling back to room temperature; empty circles – shape recovery after 1 h at room temperature). Printing parameters with higher exposure dose during printing, i.e. lower scanning speed, improve recovery rate. Full recovery of all microstructures was obtained after keeping them at room temperature for 1h.


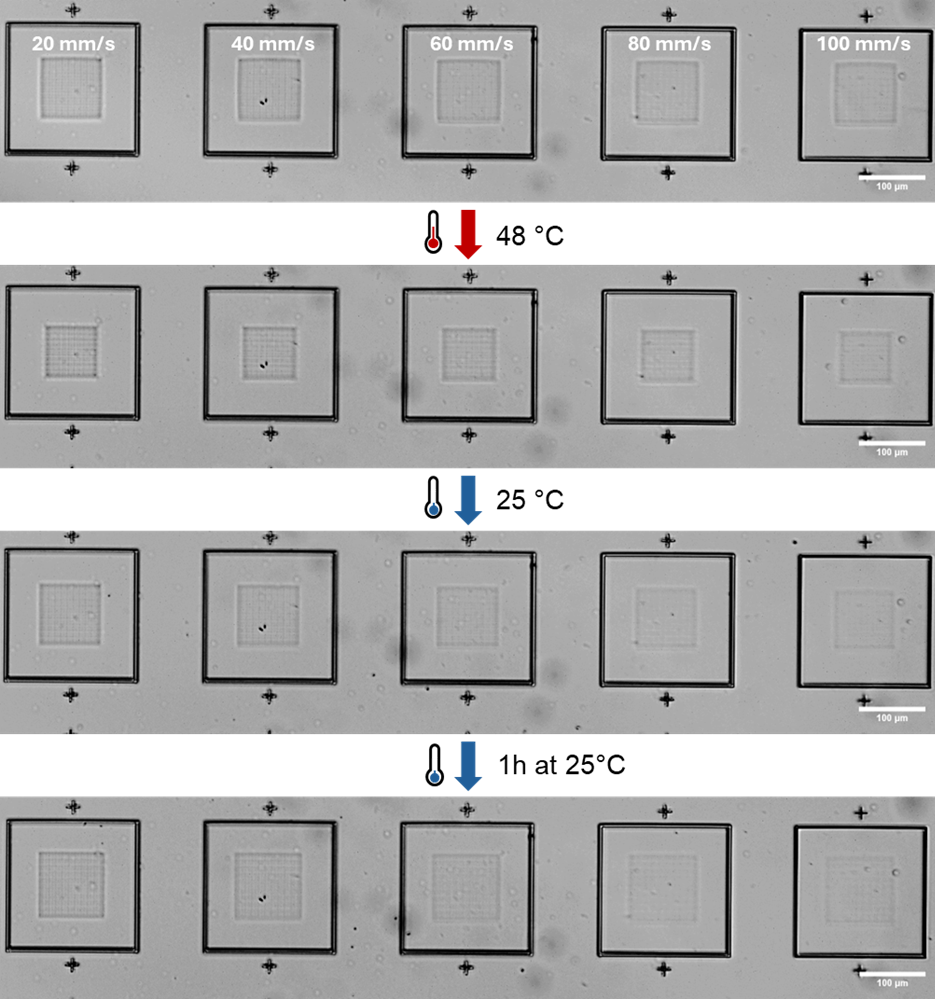


**Figure S28:** Response and recovery of 3D printed collagen microstructures with 40 mW laser power and varying scanning speed between 20 mm/s and 100 mm/s (from left to right). Full recovery of printed microstructures with higher scanning speed was obtained after keeping the sample at room temperature for 1h.

**Table S2:** Parameters employed in the stochastic molecular dynamics simulations.

| Parameter type | Parameter | GROMACS arbitrary units | Normalized units |
| --- | --- | --- | --- |
| Bonded interaction | K_bond_ | 100 | 250 k_B_T/σ^2^ |
|  | r | 2.80616 | σ(2^1/6^)/2 |
| Repulsive non-bonded Lennard-Jones interaction | ε | 0.25 | 0.1 k_B_T |
|  | σ | 2.5 | 1 σ |
|  | d_cutoff_ | 10 | 4 σ |
|  | Verlet buffer tolerance | 2·10^-4^ | ~2.5·10^-3^ k_B_T/τ |
| Angular interaction | K_θ_ | 5000 | 2000 k_B_T |
|  | θ_0_ | 180^o^ | 180^o^ |
| Langevin dynamics | m | 20000 | 1 m |
|  | 1/γ | 1000 | ~15.8740 τ |
|  | k_B_T | 2.5 | 1 k_B_T |
|  | D | 0.125 | ~1.2599 σ^2^/τ |
|  | Δt | 0.1 | ~1.5874·10^-3^ τ |
|  | No. integration steps | 10^6^–6·10^8^ steps | |
| Characteristic diffusion time | τ | ~62.996 | 1 τ |
| Closed package volume | V_CP_ | 156250 | 10000 σ^3^ |
